# 

**Published:** 1860-05

**Authors:** 


### CONTENTS.

### MAY, 1860.

### Lectures, Monographs, and Cases.

PAGE

The Physiology of the Circulation. A Course of Lectures delivered at the College of Physicians and Surgeons, New York, in the Fall Term of 1859. By John C. Dalton, Jr., M.D., Prof. of Physiology and Microscopic Anatomy. Lecture V. (With a Cut.) .......... 737

Lectures on Displacements of the Uterus. By E. R. Peaslee, M.D., LL.D., Prof. of Obstetrics and Diseases of Women and Children in the New York Medical College. Lecture III. (With Cuts.) .......... 758

Lumbar Abscess. By John S. Waggoner, M.D., of Carlisle, Cumberland Co., Penn. .......... 768

On the Resuscitation of Infants Born Still. By William C. Rogers, M.D., Green Island, Albany Co., N. Y. Second Article .......... 771

The Treatment of Paralysis of Motion. By Charles F. Taylor, M.D. .......... 782

### Monthly Summary of Medical Journalism,

By O. C. Gibbs, M.D., Frewsburg, N. Y.

Dysentery, 784; Raw Meat, 785; Quinine in Uterine Hæmorrhage, 786; Elephantiasis, 786; Hepatic Abscess, 786; Hydrocephalus, 787; Hæmaturia, 787; Scarlatina, 787; Gelseminum, 788; Indian Corn an Anti-Periodic, 788; Opium an Antidote to Belladonna, 789; Anæsthesia during Sleep, 789; Wounds of the Scalp, 789; Tonic for Dyspepsia, 790; Belladonna as an Anti-Galactic, 790; Extra Uterine Pregnancy, 791; Variola and Vaccina, 791; Podophyllin and Leptandrin, 792; Uterine Hæmorrhage, 792; Prolapsus of the Funis, 792; Injuries of the Skull, 793; Swallowing Teeth, 794; Pneumonia, 794; Valerianate of Strychnia, 795; Treatment of Gonorrhœa, 795; Ovariotomy, 795; Diphtheria, 796.

### Reviews and Bibliography.

A Practical Treatise on Fractures and Dislocations. By Frank Hastings Hamilton, M.D., Prof. of Surgery in the University of Buffalo, &c., &c. Illustrated with 289 Wood-Cuts. Philadelphia: Blanchard & Lea. 1860. Pp. 757 .......... 797

Proceedings of the American Pharmaceutical Association, at the Eighth Annual Meeting, Boston, Mass., Sept., 1859. Boston: Geo. C. Rand & Avery. 1859. Pp. 416 .......... 799

A Medico-Legal Treatise on Malpractice and Medical Evidence: comprising the Elements of Medical Jurisprudence. By John J. Elwell, M.D., Member of the Cleveland Bar. New York: John S. Voorhies. Cleveland, Ohio: Alfred Elwell & Co. 1860 .......... 801

Diseases of the Ear. By J. Toynbee, F.R.S. Blanchard & Lea. Philadelphia: 1860 .......... 803

Introductory Lectures and Addresses on Medical Subjects; delivered before the Medical Classes of the University of Pennsylvania. By George B. Wood, M.D., LL.D., &c. J. B. Lippincott & Co., Philadelphia .......... 804

On Criminal Abortions in America. By Horatio R. Storer, M.D., &c. Philadelphia: J. B. Lippincott & Co .......... 805

Braithwaite's Retrospect. Part XL. New York: W. A. Townsend & Co. 805

Ranking's Half-Yearly Abstract. Part XXX. Philadelphia: Lindsay & Blakiston .......... 806

The British and Foreign Medico-Chirurgical Review. New York: S. S. & W. Wood .......... 806

### Proceedings of Societies.

New York Medico-Chirurgical College—Morbus Coxarius; Rudimentary Tooth involuted Through the Meatus Auditorius Externus; Cystic Degeneration of the Kidneys; Action of Anæsthetics on Horses; Case of Extra-Uterine Gestation .......... 806

### Editorial and Miscellaneous.

Dr. Dalton's Lectures on *The Physiology of the Circulation*—Death of Dr. William C. Rogers—Journal de la Physiologie; Dr. Brown-Séquard—University of Maryland—Unguentum Glycerini—Formula for Pepsin Wine—A Medical Joke .......... 814

